# Establishment of a stable transfection method in *Babesia microti* and identification of a novel bidirectional promoter of *Babesia microti*

**DOI:** 10.1038/s41598-020-72489-3

**Published:** 2020-09-24

**Authors:** Dabbu Kumar Jaijyan, Kavitha Govindasamy, Jyoti Singh, Shreya Bhattacharya, Agam Prasad Singh

**Affiliations:** 1 National Institute of Immunology, Aruna Asaf Ali Marg, New Delhi, 10067 India; 2 MJKS Research LLC, Edison, NJ USA

**Keywords:** Biotechnology, Genetics, Microbiology, Diseases

## Abstract

*Babesia microti*, an emerging human pathogen, is primarily transmitted through a bite of an infected tick and blood transfusions in human. Stable transfection technique has been reported in many protozoan parasites over the past few years. However, in vivo transient and stable transfection method has not been established for *Babesia microti*. Here, for the first time, we present a method of transient as well as stable transfection of the *Babesia microti (B. microti)* in the in vivo conditions. We have identified a novel promoter of *B. microti.* We also demonstrated that *Plasmodium berghei* DHFR promoter is recognized and functional in *B. microti.* We show that BM-CTQ41297 promoter control the expression of two genes, which are present on either side and thus represents a bi-functional promoter in *B. microti.* The predicted promoter activity values using Promoter 2.0 program is higher for BM- CTQ41297 promoter than strong promoters such as β-actin, ef-1β, and many other promoters. Furthermore, we discovered a non-essential locus for the genetic manipulation of the parasite, allowing us to stably integrate foreign genes; *GFP*, *mCherry*, into the *B. microti.* The transfection using an electroporation method and genetic manipulation of *B. microti* is now achievable and it is possible to obtain transfected viable parasites under in vivo growing conditions. The growth curve analysis of transfected and WT *B. microti* are similar indicating no defects in the transgenic parasites. This study will enable other researchers in understanding the *B. microti* biology, host modulation and diverse parasite developmental stages using reverse genetics and holds great potential to identify novel drug targets and vaccine development.

## Introduction

The first case of human babesiosis was identified in 1957 and our understanding of *B. microti* is still not complete. *Babesia* is the second most common blood parasite in mammals after trypanosomes^1^. *B. microti* is primarily transmitted to human by the bite of an infected tick from genus *Ixodes* or through blood transfusion. More than 100 species of *Babesia* parasite have been documented^2–4^, and few of them cause infection in human, namely *B. microti, B. divergens, B. duncani* or *B. venatorum* (formerly known as *Babesia* sp. EU1)^5^. Human babesiosis results in 6% to 9% of fatality in normal individuals and up to 20% in immunocompromised or elderly, putting a huge economic burden on the human population^5^. The genetic manipulation of a pathogen represents a potent tool to identify and study the function of important genes for the discovery of new drug and vaccine targets. Genetic manipulation (transfection) of *B. microti* has not been reported yet owing to the intracellular life stages and complicated life cycle of the parasite. The characterization of a genetically modified parasite will greatly improve our understanding of parasite biology at various developmental stages of its life cycle.

*Babesia microti* has been suggested to have a new clade in apicomplexan parasites as revealed by the genome-wide phylogenetic analysis^6–8^. Moreover, *B. microti* is significantly distant from other *Babesia* and apicomplexan parasites suggesting that the previously established methods of transfection for other species of *Babesia* such as *B. gibsoni*^9,10^, *B. bovis*^11–17^, *B. bigemina*^11,12^, *B. ovata*^18^ and apicomplexan parasites may not work for this particular parasite^7,8^. Furthermore, *B. microti* parasite has smallest nucleus among all the apicomplexan parasites owing to the impediment of successful transfection of this parasite^6^. A transient transfection of *B. microti* in in vitro condition has been reported recently using beta actin promoter driving the expression of a luciferase gene^19^. Since long-term in vitro culture has not yet been established for *B. microti,* therefore, this method may not be suitable to perform stable transfection of parasite as mentioned in the published paper. Moreover, in vitro and in vivo growing parasites behave differently in their response to drugs, vaccine, and adaptation.

The transfection system has been established in other apicomplexan parasites such as *Theileria annulata*^20^, *Toxoplasma gondii*^21^, *Cryptosporidium parvum*^22^, *T. parva*^23^, *Plasmodium falciparum*^24^ and other *Plasmodium* species (*P. berghei*, *P.yoelii*, *P.knowlesi*). These methods cannot be implicated in *B. microti* due to its different life stages and its smallest size. Moreover, purification of *B. microti* via density gradient centrifugation is very challenging, which is a requirement in other methods. In this study, we established an efficient method of stable as well as transient transfection in *B. microti* growing in in vivo conditions using a combination of reporter gene assays and electroporation approach. Our transfection method has many advantages over a recently reported in vitro transient transfection in *B. microti*. Expressing a fluorescent marker in the parasite will be advantageous in locating the parasite in a tick vector and host cells.

## Results

### Identification and bioinformatic analysis of BM-CTQ41297 promoter

The success of an efficient transfection method critically depends on the selection of a strong promoter, and a suitable strategy for DNA transfection. We have achieved a first-ever reported stable genetic manipulation of *B. microti* parasite. We used green fluorescent protein (GFP) as a transfection marker in parasite because of easy identification and visualization of GFP expressing parasites under a fluorescence microscope, followed by isolation using fluorescence-activated cell sorting (FACS). First, we identified a BM-CTQ41297 promoter in *B. microti* parasite (https://protists.ensembl.org). The BM-CTQ41297 promoter primarily controls the transcription of *BMR1_03g03485* gene, which encodes for a hypothetical protein named as CTQ41297. The promoter activity values for BM-CTQ41297 promoter is higher (1.07) than other constitutive promoters such as human ef1α (0.609), human ef1β (0.713), and CMV (0.549) promoters as analyzed by a promoter prediction server 2.0. The value of promoter strength for these promoters is shown in Table 1. *B. microti* promoter (1969 nucleotides) was further analyzed for a strong promoter activity region using a bioinformatic tool (https://www.softberry.com/berry.phtml?topic=bprom&group=programs&subgroup=gfindb). This tool is reliable and it has been used in more than 800 publications. This tool is used to predict a region in the nucleotide sequences, which are predicted to be a promoter sequence. The bioinformatic analysis shows that a nucleotide sequence between 700 bp and 1300 bp region of parent 1969 nucleotide sequence may have a strong functional promoter activity. Table 2 describes the parts of BM-CTQ41297 promoters that are predicted to have promoter activities. These analyses show that transcription factors binding site score is higher for the nucleotides 778–1200 bp.

**Table 1:** Activity score for various promoters predicted by Promoter 2.0 server.

Promoter name	Predicted values by Promoter 2.0 server	Accuracy of prediction
BM-promoter	1.07	A score > 1 represents an accuracy of 95% true
Human ef1α	0.609	A score of 0.5–0.8 have an accuracy of 65% true
Human ef1β	0.713	A score of 0.5–0.8 have an accuracy of 65% true
CMV promoter	0.549	A score of 0.5–0.8 have an accuracy of 65% true

**Table 2:** Various regions of BM-CTQ41297 that have high transcription factor binding and promoter threshold score (software has an accuracy of 80% true and threshold value is 0.20).

BM-CTQ41297 regions	First nucleotide of section	Last nucleotide of section	Promoter threshold score
a	796	833	8.35
b	1166	1203	5.64
c	1698	1733	5.43
d	418	457	4.44
e	33	70	2.09

BM-CTQ41297 regions	Promoter position	Position of 1st nucleotide	Transcription factor binding score
f	833	778	16
g	833	781	15
h	833	821	15
i	1,203	1,194	17
j	1,203	1678	17
k	1733	1677	14
l	1733	1678	17

### Establishment of an in vivo transient transfection method of *B. microti*

**Figure 1:**
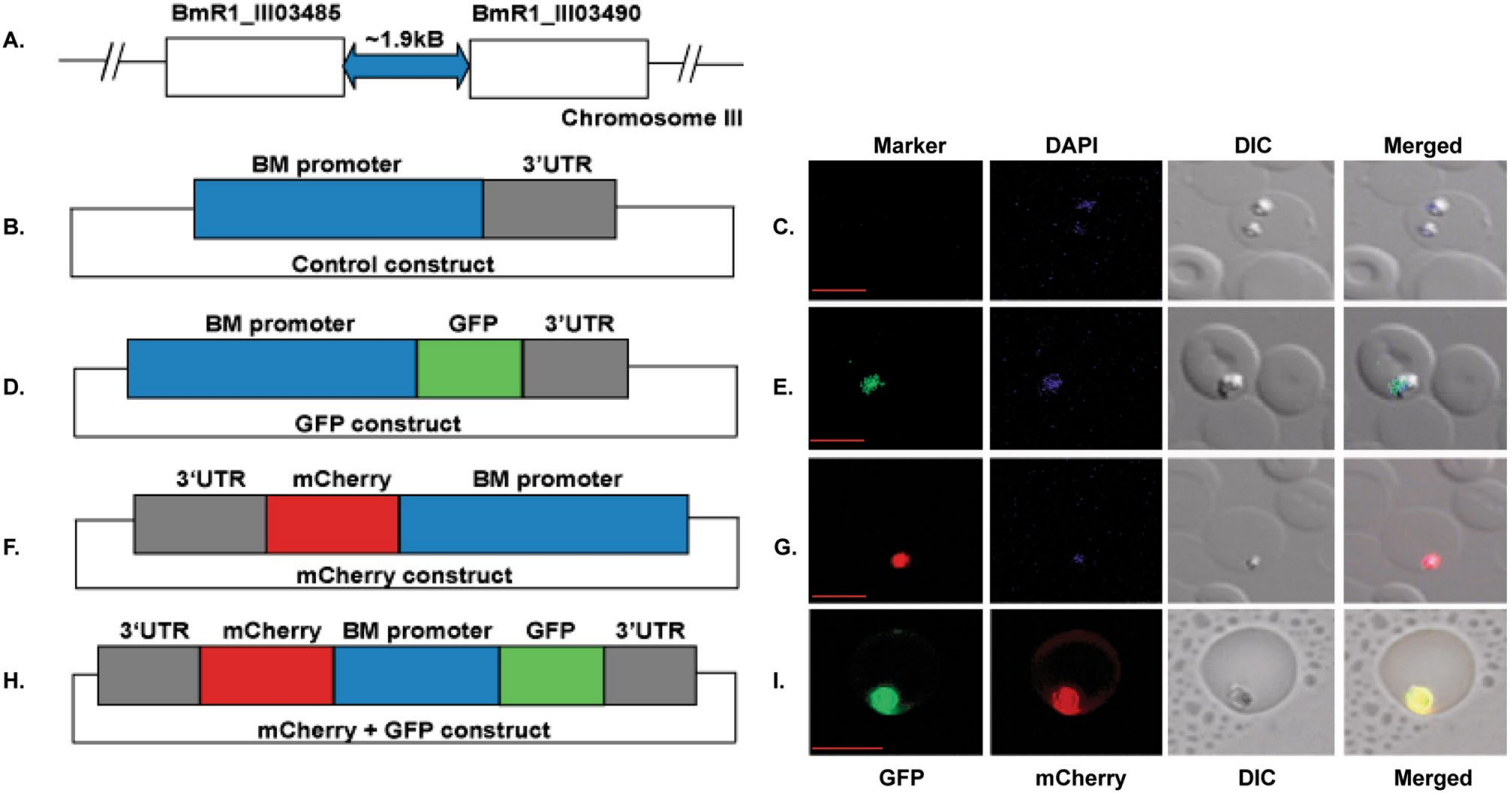
Transient transfection of *B. microti* parasite. (**A**) Genomic analysis of a BM-CTQ41297 promoter in the parasite. (**B**) Pictorial representation of a construct map for control plasmid. (**C**) Images represent the *B. microti* parasites transfected with the control plasmid. DAPI staining corresponds to the nucleus of the parasite within RBC. Merged image represents the overlap of all images. Scale bar represents 5 μm. (D) Map of a transient transfection construct containing *GFP* gene. (**E**) Green flourescence corresponds to the transfected parasite expressing GFP, DAPI staining represents the nucleus of the parasite, and DIC image showing a parasitized RBC. Merged image represents the overlap of all images. Scale bar represents 5 μm. (**F**) Map of a mCherry construct used for parasite transient transfection. (**G**) On the right side red flourescence corresponds to the transfected parasite expressing mCherry. DAPI staining (Blue) corresponds to the nucleus of the parasite, and DIC is showing a parasitized RBC. Merged image represents the overlap of all images. Scale bar represents 5 μm. (**H**) Construct map of a plasmid containing *GFP* and *mCherry* gene on either side of a BM-CTQ41297 promoter. (**I**) Green and red fluorescence signal correspond to the same parasite expressing GFP and mCherry. DIC image represents the parasitized RBC and merged is the overlap of all the images. Scale bar represents 5 μm.

An extensive genomic location analysis of BM-CTQ41297 promoter revealed that it is flanked by two genes which are present at each end as shown in Fig. 1A and suggested a possibility of it being a bifunctional promoter. We prepared an episomal construct, in order to check the efficacy of a BM-CTQ41297 promoter to express a reporter gene in *B. microti* parasite. In control experiments, the *B. microti* parasites were transfected with an episomal construct without any GFP gene (Fig. 1B) and were GFP negative in fluorescence microscopic analysis (Fig. 1B,C). The test episomal construct contained a BM-CTQ41297 promoter, a *gfp* gene at downstream region of the promoter and a 3′UTR of *BMR1_03g03490* gene. The details of the episomal construct are shown in Fig. 1D. The test episomal construct electroporated parasites were GFP positive as confirmed by fluorescence microscopy and shown in Fig. 1E. It confirmed that BM-CTQ41297 promoter could be used to express a reporter gene in *B. microti* parasite.

Next, we used *mCherry* (a modified version of GFP, which gives red fluorescence) gene at an upstream location of the BM-CTQ41297 promoter in our second test episomal construct for transfection of *B. microti* parasite (Fig. 1F). We wanted to check if a BM-CTQ41297 promoter could express a reporter gene inserted at the upstream region of the promoter in parasite. The fluorescence microscopic analysis of transfected parasites confirmed that *mCherry* gene when placed at an upstream of BM-CTQ41297 promoter was expressed in the electroporated parasite as compared to the control (Fig. 1G). Taken together, results confirmed that BM-CTQ41297 promoter can express a foreign gene located either downstream or upstream of the promoter. In a nutshell, we have optimized the conditions to successfully introduce and express reporter genes into *B. microti.*

### BM-CTQ41297 is a bidirectional promoter

In *B. microti,* genomic location of BM-CTQ41297 promoter, is flanked by two different genes (Fig. 1A). We were curious to know whether a BM-CTQ41297 promoter has a bifunctional activity or not. Therefore, we cloned a BM-CTQ41297 promoter in pBSK vector with *mCherry* and *GFP* genes on either side along with UTR regions in the same vector as described in Fig. 1H. Parasites transfected with GFP-mCherry dual construct plasmid expressed GFP as well as mCherry markers as confirmed by the fluorescence microscopy (Fig. 1I). Our results confirmed that BM-CTQ41297 promoter has a bidirectional promoter activity and can be used to express two markers simultaneously in *B. microti* (Fig. 1I). This is a first-ever that a novel bidirectional promoter is identified in *B. microti* parasite and demonstrated its use. The high efficacy of BM-CTQ41297 could be attributed to its native resident promoter of *B. microti*, bidirectional activity, and high recognition as well as binding to the transcription factors suggested by bioinformatic analysis (Table 1 & Table 2).

#### A promoter from Plasmodium is recognized in B. microti

**Figure 2:**
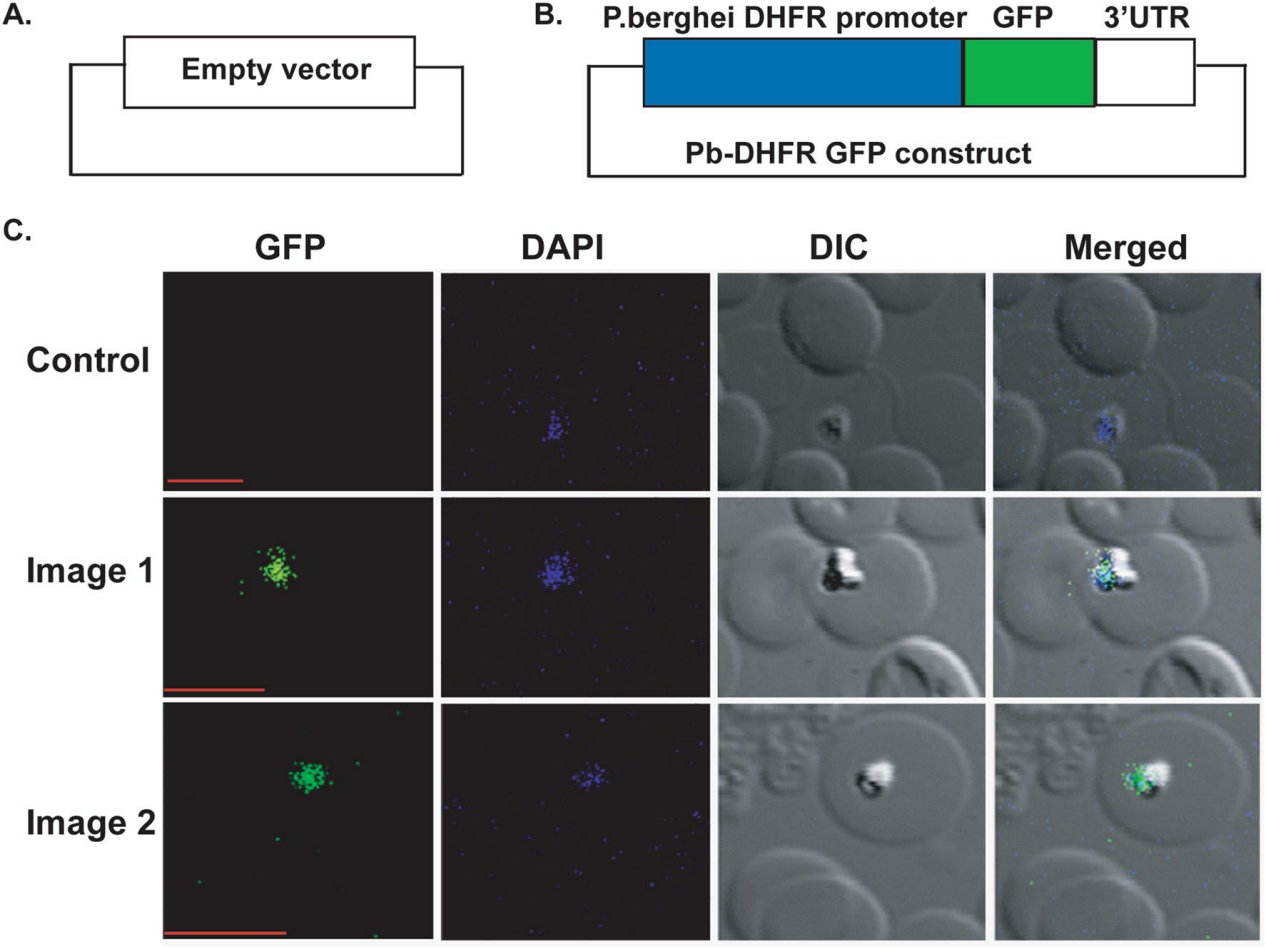
*Plasmodium berghei* DHFR promoter is recognized by the *B. microti* parasite. (**A**) The map of an empty plasmid construct used as a control. (**B**) The map of a transient transfection construct containing a Pb-DHFR promoter and a *GFP* gene. (**C**) The green image represents the parasite expressing GFP and DAPI represent the parasite nucleus stained with DAPI. DIC is showing the infected RBC. Control represents the parasite transfected with control plasmid without any DHFR promoter and *GFP* gene. Scale bar represents 5 μm.

The *Plasmodium berghei* DHFR (Pb_DHFR) promoter is well known for its strong activity and application for transfection in *Plasmodium* species. We hypothesized, that Pb_DHFR promoter might be recognized by transcriptional machinery in *B.microti* parasite. To test our hypothesis, we prepared a construct that contains a ~ 2.20 kb upstream sequence of Pb_ANKA-DHFR promoter and a *GFP* gene along with ~ 600 nucleotides from the 3′UTR of *BMR1_03g03490* (Fig. 2B). Twenty-four hours post-electroporation transfected parasites were found to be GFP positive as compared to the control (Fig. 2A,C). Our results confirmed that Pb_DHFR promoter is recognized by *B. microti* parasites albeit with less efficiency as compared to *B. microti* BM-CTQ41297 promoter. This indicates that the affinity of transcription factors in *B. microti* is less for Pb-DHFR promoter. This is also the first report that a promoter from *Plasmodium* could be used in the *B. microti.* All the above results strongly suggest that we have optimized and established various conditions for the transient transfection of *B. microti* parasite that form the foundation of a stable transfection method.

### BM-CTQ-41297 outcompetes other strong promoters

**Figure 3:**
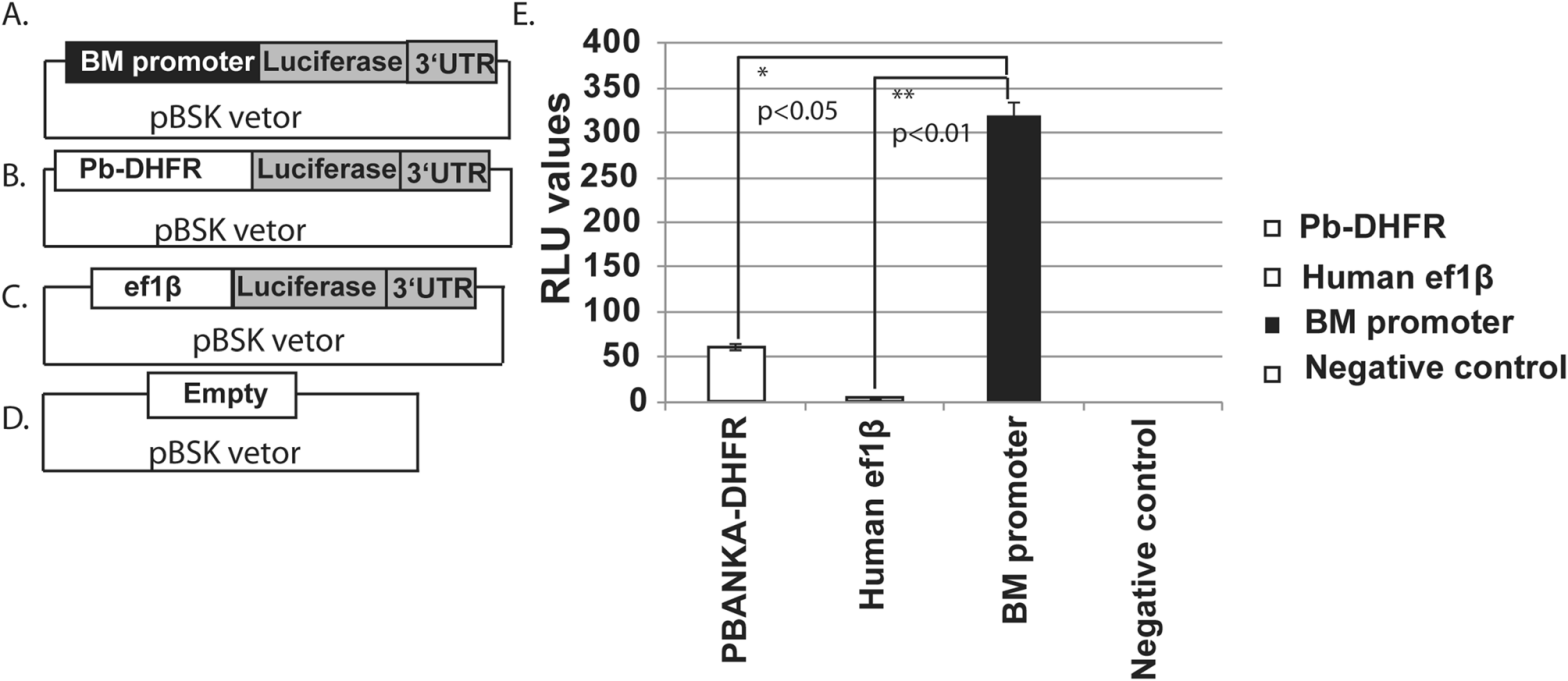
Luciferase expression in the *B. microti* parasite. (**A**) A map of plasmid containing luciferase open reading frame (ORF) under the BM-CTQ41297 promoter. (**B**) A map of plasmid containing luciferase ORF under the Pb-DHFR promoter. (**C**) A map of plasmid containing luciferase ORF under the ef1β promoter. (**D**) A map of an empty plasmid used as a control. (**E**) Luciferase activity measured as RLU values in transiently transfected parasites with respective construct. Each bar represents a mean RLU and standard deviation from 3 independent experiments. The statistical analysis was performed by student t-test. The p-values were *p < 0.05, **p < 0.01.

To determine the promoter strength, we measured the relative luciferase activity for various promoters in the transfected parasites. The transfection constructs containing luciferase gene under the control of BM-CTQ41297 promoter (Fig. 3A), Pb-DHFR (Fig. 3B), ef1β promoter (Fig. 3C) and empty vector (Fig. 3D) were electroporated into the *B. microti* parasite. The luciferase activity was measured 36 h post-transfection and results confirmed that BM-CTQ41297 promoter was transcribing luciferase expression at a higher level than ef1β and Pb-DHFR promoters (Fig. 3E). We observed that luciferase activity was approximately 6 folds higher in case of BM-CTQ41297 promoter when compared to Pb-DHFR promoter (Fig. 3E). These results strongly suggest that BM-CTQ41297 promoter can drive the expression of target gene at higher rate in *B. microti*.

### Establishment of an efficient method of stable transfection of *B. microti*

**Figure 4:**
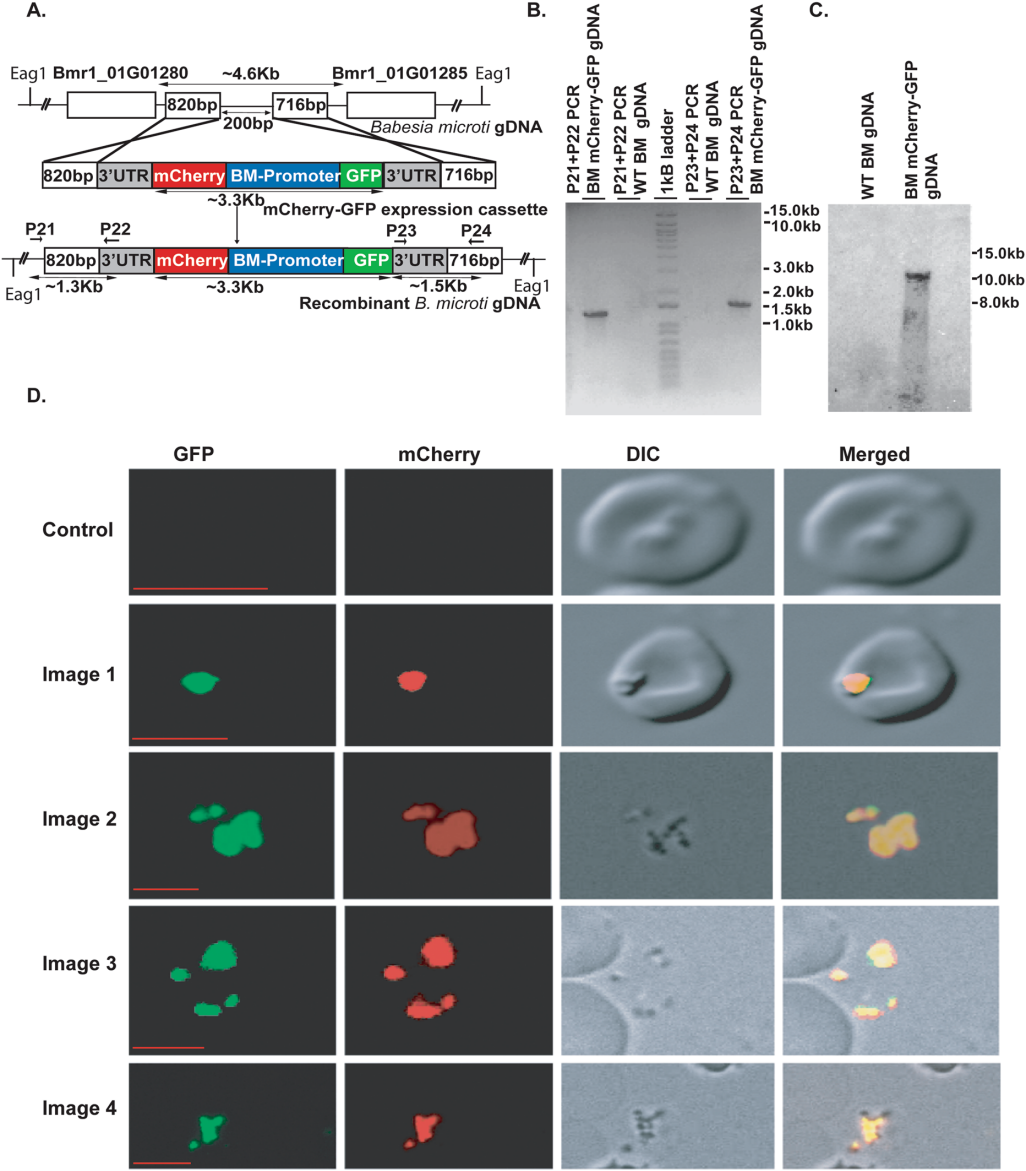
Stable transfection of the *B. microti* parasite. (**A**) Pictorial representation of a double homologous recombination strategy used for creating a recombinant *B. microti* parasite expressing *GFP* and *mCherry* gene in a same parasite. (**B**) Diagnostic PCR. The primer P21 and P22 is specific for the 5′ integration event and it should not give any PCR product on WT gDNA. The primer P23 and P24 is specific for the 3′ integration event. A PCR product of right size in the diagnostic PCR confirmed the integration of GFP-mCherry cassette at the right locus in *B. microti* parasite. Image shown is cropped and pixel inverted for better visualization and printing. Original image is provided in the supplementary file for clarity and comparison. (**C**) Southern blot analysis of transfected parasites. The DIG labeled GFP probe was used to detect the integration event in the digested gDNA. A band of ~ 10.5 kb was detected in the transgenic *B. microti* parasite. Wild-type parasites do not have the gene for GFP hence no bands are expected. Image shown is cropped and color converted to grey scale for better visualization and printing. Original image (color) is provided in the supplementary file for clarity and comparison. (**D**) Confirmation of a stable transfection in *B. microti* by fluorescence microscopy of the parasites expressing the *GFP* and *mCherry* gene. Green and red images correspond to the parasite expressing GFP and mCherry gene within a stably transfected parasite. Merged represents the overlap of all the images. Scale bar represents 5 μm.

The genetic manipulation using reverse genetics is a powerful tool to study the function of a gene in the parasite. To perform a stable integration of GFP and mCherry markers in *B. microti* parasite, we prepared a targeting construct containing a bifunctional BM-CTQ41297 promoter along with *GFP* and *mCherry* reporter genes as shown in Fig. 4A. The targeting construct was linearized with SapI restriction enzyme and electroporated into the *B. microti* parasites. Electroporated parasites were immediately injected into the mice and later on analyzed by the fluorescence microscopy, Southern blot and diagnostic PCR methods. Clonal population of transgenic parasite was analyzed by diagnostic PCR and Southern blot analysis. The diagnostic PCR, as well as Southern blot analysis, confirmed the integration of reporter genes at the right locus in the parasite genome (Fig. 4B,C). In the diagnostic PCRs, P21 and P22 represent the primers (Table 3) used to show successful 5′ integration, and similarly, P23 and P24 (Table 3) represent the primer pair that was used to demonstrate 3′ integration. The diagnostic PCR results confirm the integration of reporter genes in the *B. microti* parasite at right locus by showing PCR products of expected sizes (Fig. 4B). A Southern Blot analysis of wild type (WT) and transgenic *B. microti* parasite genomic DNA (gDNA) detected the expected band size of ~ 10.5 kb from the transgenic parasite using a GFP probe, but not in the in the WT parasite gDNA, which does not contain the GFP (Fig. 4C). Moreover, as shown in the Fig. 4D expression of both GFP as well as mCherry proteins was observed in the same parasite, which suggested a stable transfection in *B. microti* parasite. We did not incorporate any drug selection marker, therefore, we used a fluorescent activated cell sorting (FACS) to sort the GFP and mCherry positive parasites.

**Table 3:** List of primers used in this study.

Primers	Sequence
P1-XHO1	ATT CTCGAG TGCCA TTGTTATTCA CGCTAG
P2-HINDIII	GCC AAGCTT TTTTCTAACATTCAAGAGGCTGC
P3-HINDIII	GCCAAGCTTATGAGTAAAGGAGAAGAACTTTTC
P4-BAMH1	ATTGGATCCTTATTTGTATAGTTCATCCATGC
P5-BAMH1	ATTGGATCCACATGAC ACATACTTGG TGCGT
P6-NOT1	ATT GCGGCCGCACCATGTCACAATACAACATATTATG
P7-XBA1	CGGTCTAGAATGGTGAGCAAGGGCGAGGAGGAT
P8 SMA1	ATTCCCGGGCTACTTGTACAGCTCGTCCATGCC
P9-SMA1	ATTCCCGGGGTATATCGGGGAGAGCGGCAACGT
P10-SMA1	ATTCCCGGGGCTACAAGCTGGTGTGCTTTACC
P11-XHO1	ATTCTCGAGATGGTGAGCAAGGGCGAGGAGGAT
P12-XHO1	ATTCTCGAGGCTACAAGCTGGTGTGCTTTACC
P13-XHO1	ATTCTCGAGAAGTGTGTTATGAATATTTTAAG
P14-ECOR1	GCCGAATTCTTTGTAACATTTAGGTGTGT
P15-ECOR1	GCCGAATTCATGAGTAAAGGAGAAGAACTTTTC
P16-BAMH1	ATTGGATCCTATTTGTATAGTTCATCCATGC
P17-KPN1	ATTGGTACCCCTTCTCCAC TGCTACTTTT TAG
P18-XHO1	ATTCTCGAGATCATATTCGGACATAGAAATAA
P19-NOT1	ATTGCGGCCGCAGTAAGTGCT AAATGACGAT TTC
P20-SAC1	ATTGAGCTCCAAATCCACATACTTCTATGCTC
P21	ATTCTTTAAA TGCATAAATA ATAATAG
P22	TGGTAGCACCTTGTCATGCTTG
P23	GACCACATGGTCCTTCTTGAGT
P24	ACTGTATATTATATGGAATTATTCTA
P25-HindIII	GCC AAGCTTATGGAAGACGCCAAAAACATAAAG
P26-BamH1	ATTGGATCCTTACACGGCGATCTTTCCGCCCTTCTTG
P27-Sal1	ATTGTCGACCCTTTTCTCAGTTTCATTGACCA
P28-HindIII	GCC AAGCTTGGTCTCCGAAACCCATGGTGTCGG

### Transgenic *B. microti* parasites do not show any growth defects

**Figure 5:**
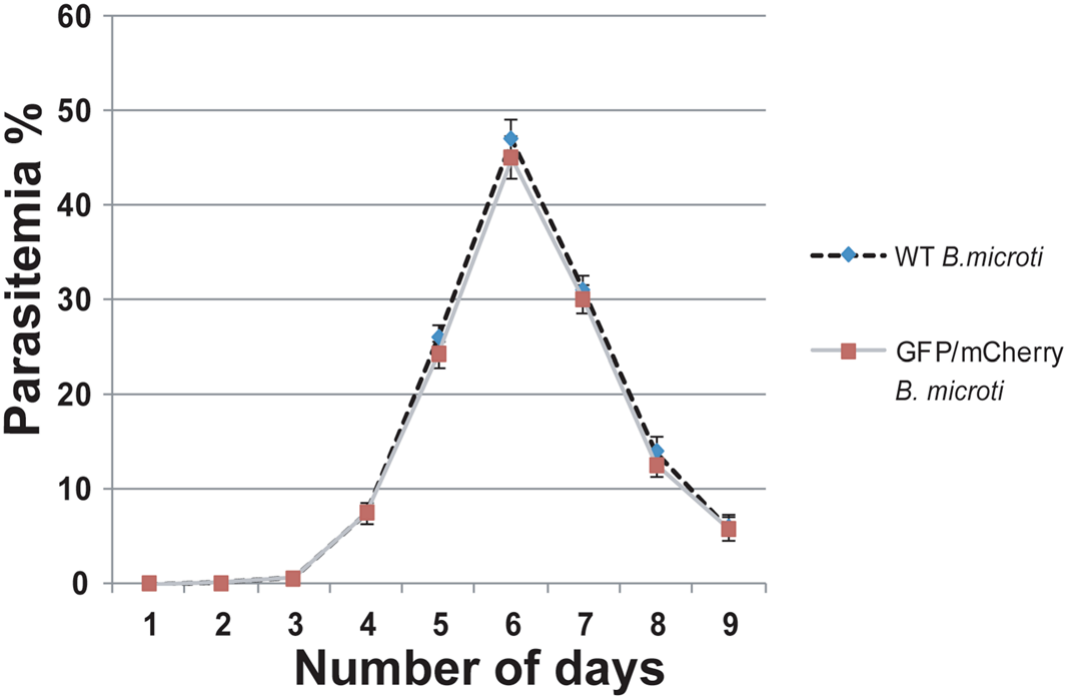
Growth curve analysis. Pre-patent period analyses for the transgenic and wild type *B. microti* parasites. The growth of wild type and transgenic *B. microti* parasites are similar. Each data point represents an average parasitemia of five mice with SD. The p-value (p = 0.5) was calculated using t-test. A p-value of p > 0.05 represents statistically not significant.

Finally, we performed a growth curve analysis of transgenic *B. microti* parasites expressing reporter genes using blood-stage parasitemia examination. Parasitemia is the quantitative measurement of parasites growth in the blood. We found transgenic parasites were similar in growth as compared with the wild type parasites (Fig. 5). The prepatent period of WT and transgenic parasite was similar (Table 4) as determined by the first emergence of parasites in infected animals. These results demonstrated that we selected a right locus for a stable transfection of the parasite and transgenic parasites were growing normally even after successful integration of fluorescent markers.

**Table 4:** Prepatent period for BM-WT and BM-GFP/mCherry parasites.

Parasite	% Parasitemia (Average ± SD)		Delay in pre-patent period on day 1
	(Day 1)	(Day 5)	
*Babesia microti-WT*	**0.005** ± 0.001	**25.9** ± 1.28	NA
*Babesia microti*-GFP	**0.005** ± 0.001	**24.3** ± 1.35	0.0 (p = 0.134)^$^

*NA* not applicable.

^$^Students t-test.

## Discussion

*Babesia microti* is an emerging Apicomplexan parasite that causes babesiosis in human and transmitted through the bite of an infected tick that belongs to the genus *Ixodus*. *B. microti* parasite can also be transmitted through the blood transfusion as asymptomatic blood donor can donate blood, and thus represent a vital problem for the military hospitals and blood bank^25^. It has been suggested that *B. microti* can be transmitted to a developing fetus in an infected pregnant woman and may have severe consequences^26^. Babesiosis cases in humans are increasing with time and represent a serious threat to the human population. Furthermore, a coinfection of *B. microti* and *Borrelia* in humans, represent a severe health issue^27,28^. Therefore, there is an urgent need to develop new tools to prevent new infection and transmission of parasite. More specifically, there is an urgent need to develop an efficient method of transfection for genetic manipulation particularly in *B. microti*. The genetic manipulation of a microorganism represents a valuable tool to study the function of a gene at different developmental stages in the parasite life cycle, identification of novel targets for drug discovery and vaccine development. A GFP expressing parasite is advantageous for the drug screening and in vivo tracking of the parasite location during infection. Studies have shown that transfection in *B. bovis,* to study the gene function, were helpful in developing vaccine against bovine babesiosis^29,30^. Therefore, a transgenic *B. microti* parasite will also be helpful in study of gene function and vaccine development. A recently published in vitro transient transfection is not feasible for the in vivo growing *B. microti* parasite as described by the author. The different adaptations of in vitro and in vivo growing parasites respond differently to the drugs, and other therapeutics. Moreover, continuous *in-vitro* growing parasite loses their capacity of transmission through the tick vector. Furthermore, a continuous in vitro culture for long-term has not yet been developed for *B. microti*, therefore, there was an urgent need to identify a new method of transfection that can be used in in vivo conditions. The establishment of a transfection method in *B. microti* parasite has opened several research areas of intense interest to investigate various research problems including identification of the critical genes associated with a parasite life stages, tick-parasite interactions, comprehensive analysis of sporogonic cycle, and host-parasite interactions. We have established for the first-time a successful method of in vivo transient and stable transfection in *B. microti* parasite by expressing *GFP* and *mCherry* reporter genes under the control of a novel promoter. First, we identified right promoters and checked their efficacy in the expression of reporter genes in *B. microti*. Next, we established a method for the transient as well as stable transfection in the parasite using constructs containing the selected promoter.

We identified sets of promoters that can be used in the transfection of *B. microti* parasite. We identified and characterized a BM-CTQ41297 promoter in *B. microti* parasite. This promoter is located on the chromosome-III. The bioinformatic analysis of BM-CTQ41297 has identified several regions listed in Table 1 which may engage with transcription factors and in RNA polymerase recognition that might lead to strong promoter activity. Genomic location of this promoter is very unique in presence of two genes at each end of the promoter. Our result shows that the BM-CTQ41297 promoter is a bidirectional promoter and can express two proteins simultaneously. A bidirectional promoter is advantageous in several ways such as expression of two reporter genes, one at either end of the promoter. In another scenario, a bidirectional promoter can be used to express a reporter and a drug selection marker.

The 2A self-cleaving peptide (2A) is an oligopeptide (usually 19–22 amino acids) located between two proteins in some members of the picornavirus family^31^. The 2A self-cleaving peptide of FMDV might undergo self-cleavage to generate mature viral proteins by a translational effect that is known as “stop–go” or “stop-carry”. The cleavage site is located between the last glycine of its C-terminal and the first proline of the 2B downstream protein. Porcine teschovirus-1, 2A (P2A-GSG) exhibits the highest cleavage efficiency. Using P2A-GSG peptide, a *B. microti* bifunctional promoter could be used to express 3 or 4 different reporter genes or a combination of drug makers and reporter genes^32^.

We used BM-CTQ41297 promoter for a first ever developed stable transfection method in the *B. microti* parasite. Moreover, a DHFR promoter from the other Apicomplexan species, the *Plasmodium berghei* was tested in this study and found to be recognized in the *B. microti* parasite with lesser efficiency as compared to the BM-CTQ41297 promoter. Furthermore, the optimal expression of *GFP* gene with Pb-DHFR promoter was observed at 4 days post-transfection, which indicates a weak recognition of a heterologous promoter. Our results also suggested that the transcriptional machinery in *B. microti* could recognize a foreign promoter from other Apicomplexan parasite such as *Plasmodium*. These results also hint at the existence of some common transcription machineries in these two parasites and a possibility of a common drug target.

This is the first time a novel bidirectional promoter in *B. microti* parasite have been identified by us and used it for the establishment of a transfection procedure in the parasite. After the establishment of appropriate conditions for the transient transfection in *B. microti* parasite, we performed a stable transfection of the parasite using double homologous recombination method. Our results demonstrated that we have identified right conditions to achieve a stable transfection of the *B. microti*. We also found a genomic locus for the genetic manipulation of the parasite, which is located in the inter-genic region of *BMR1_01G01280* and *BMR1_01G01285* genes. This locus can be used to integrate any foreign gene into *B. microti* parasite without affecting the parasite growth. An insertion of a foreign gene at this locus did not affect the parasite life cycle and transgenic parasites were similar in growth as compared to the parental parasite.

The development of an efficient transfection method in *B. microti* will springboard the foundation of new drug discovery for the babesiosis treatment. The transfection procedure can be implemented in unraveling the function of various genes including transcription factors, metabolic genes, promoters, kinases, ion channels, housekeeping and apicoplast genes by reverse genetics using gene knockout study to identify novel drug candidates. The transfection method established here can be employed to knockout a particular gene from the genome of *B. microti* followed by the phenotypic analysis of knock out parasite to identify new drug targets. The GFP/mCherry expressing parasite is very useful in screening of antiparasitic compounds by using FACS to determine parasitemia, parasite invasion and replication after drug treatment. *Plasmodium* expressing a reporter gene has been used in high throughput drug screening previously^33^ suggesting the applications of current transfection procedure. The transfection methods have been used to develop genetically attenuated parasite (GAP) vaccines in the *Plasmodium* indicating another potential application of an efficient transfection method in developing a vaccine^34,35^. A GFP expressing parasite can be easily imaged by microscopy after the drug treatment will lead to a potential screening of compounds and eliminate the antibody staining to identify parasites after drug administration. A luciferase-expressing parasite has been used in drug screening under in vitro as well as in in vivo conditions^36^.

Transfection method developed by us will be very useful to characterize the unidentified stages of the parasite life cycle in tick stage. Moreover, our study will be useful for identifying novel drug and vaccine targets. Furthermore, our study has applications in generating a live attenuated parasite vaccine though the genetic engineering of the parasite. There are many other Apicomplexan parasites where a stable transfection method is lacking. Taking clues from our findings, we anticipate that transfection of other parasite can be achieved through a right combination of promoter, fluorescent markers and efficient electroporation method.

## Material and methods

### Experimental animals

Experiments related to animal were performed according to the protocol (NII/488/18) approved by CPCSEA controlled Institutional Animal Ethics Committee (IAEC), National Institute of Immunology (NII), India. The general care of the experimental animals for this study was in accordance with animal use guidelines for laboratory animals and in compliance with the Animal Welfare Act (Prevention of Cruelty to Animals Act 1960, Wildlife division, Ministry of Environment and Forest, India). The C57BL/6 or BALB/c mice aged 4–6 weeks were used for parasite propagation and infection. The animals were obtained from NII’s in house breeding facility, India.

### Construct preparation

#### Promoter selection

*B. microti*-CTQ41297 promoter that we selected is located in chromosome-III. We named this promoter as a BM-CTQ41297 promoter and it comprises a nucleotide sequence of ~ 1.9 kb upstream of the start codon of *BMR1_03g03485*. This promoter controls the expression of a conserved protein named as CTQ41297, which is predicted to be a trafficking protein particle complex subunit-1, putative (BET-5), The gene (*BMR1_03g03485*) is present on chromosome-III and encoded in the reverse direction. We selected the BM-CTQ41297 promoter for our study owing to its predicted high promoter activity value by a Promoter 2.0 prediction server (Table 1). Our selection criteria for choosing a strong promoter was to compare the predicted putative values for BM-CTQ41297 promoter to other strong known promoters such as human ef1α, human ef1β, *B. bovis* ef1α, or CMV (Cytomegalovirus, Addgene seq. #vdb/6228) promoter. The predicted promoter activity value for the BM- CTQ41297 promoter was higher than the predicted value for other known strong promoters as mentioned earlier. The various regions of the BM-CTQ41297 promoter sequence predicted to have strong promoter activity are shown in Table 2.

#### DNA constructs for the transient transfection of B. microti

##### GFP construct

To establish a method of the transfection in *B. microti* parasite, we prepared a plasmid construct that contains a GFP expression cassette having a *GFP* gene under the control of a *B. microti* promoter (BM- CTQ41297) and 3′UTR of *BMR1_03g03490* gene (~ 627 bp). The BM-CTQ41297 promoter (~ 1.9 kb) was amplified by the polymerase chain reaction (PCR) from Bm-gDNA using P1-XhoI and P2-Hind-III primers and a Pfu polymerase. PCR product was cloned into the pBSK(+) plasmid at XhoI and Hind-III restriction sites. Next, a *green fluorescence protein* (*GFP*) gene was cloned between Hind-III and BamHI restriction sites. The *GFP* gene was amplified using primers P3-Hind-III and P4-BamHI from a GFP expressing Pb_ANKA parasite (Pb-GFP con) gDNA. At 3′end of a *GFP* gene, ~ 627 nucleotides from 3′UTR of *BMR1_03g03490* gene was cloned for proper termination of GFP-mRNA. The 3′UTR of *BMR1_03g03490* was amplified by PCR from BM-gDNA using P5-BamHI and P6-NotI primers and cloned at BamHI and NotI restriction sites into the pBKS plasmid. We named this construct as plasmid **T1**, which contains *GFP* gene under the control of a BM- CTQ41297 promoter. A complete list of primers used in this study is described in Table 3.

##### mCherry construct

We cloned a *mCherry* gene along with ~ 627 bp from 3′UTR of the *BMR1_03g03490* gene at upstream of the BM-CTQ41297 promoter into a transfection plasmid at Xho-I site. The mCherry gene was amplified from a Pb_ANKA parasite gDNA containing *mCherry* gene (Pb_mCherry, unpublished, A gift from Dr. Arun K. Kota, India) using primers P7-XbaI and P8-SmaI and was cloned into the pUC19 vector at XbaI and SmaI site to generate pUC19-mCherry plasmid. The *BMR1_03g03490* 3′UTR (~ 627 bp) was amplified from *B. microti* gDNA using primer P9-SmaI and P10-SmaI and was cloned into the pUC19-mCherry plasmid at Sma-I site. Thereafter, the *mCherry* gene with 3′UTR was amplified using primer P11 and P12 and cloned into the pBSK-BM construct at Xho-I site. This construct contains a *mCherry* gene at 5′ end of BM-CTQ41297 promoter. This construct was named plasmid **T2.**

#### Bifunctional promoter assay construct preparation

The mCherry cassette was amplified from gDNA of Pb-mCherry parasite (a gift form Arun K. Kota) using P11 and P12 and was cloned into the GFP transfection construct (plasmid T1) at XhoI site. Therefore, this construct contains a GFP gene at downstream and a mCherry gene at upstream region of a BM-CTQ41297 promoter. We named this construct as plasmid **T3.**

#### Plasmodium berghei DHFR promoter construct

To test an interspecies promoter from another apicomplexan parasite we cloned a ~ 2.2 kb region of 5′UTR of the Pb_DHFR-TS gene into a pBSK ( +) vector between XhoI and EcoRI restriction sites. The 5′UTR of the Pb_DHFR gene was amplified from Pb_ANKA gDNA using primers P13-XhoI and P14-EcoRI**.** Next, we cloned a GFP gene between EcoRI and BamHI in pBSK vector containing DHFR promoter from Pb_ANKA. The GFP gene was amplified using primers P15-EcoRI and P16 BamHI. At 3′end of *gfp* gene, ~ 627 nucleotides from 3′UTR of *BMR1_03g03490* gene were cloned for proper termination of *gfp*-mRNA. The 3′UTR was amplified by PCR from Bm-gDNA using P5-BamHI and P6-NotI primers and cloned at BamHI and NotI restriction sites into the pBSK plasmid. This construct was named as plasmid **T4.**

#### Promoter activity analysis using luciferase assay

The BM-CTQ41297 (~ 1.9 kb), and Pb_ANKA-DHFR-TS (~ 2.2 kb), promoters were cloned at XhoI and Hind-III sites of a pBSK vector. The human ef1β promoter sequence (~ 1.6 kb) from the upstream region of the start codon of the ef1β gene was amplified using primers P27-SalI and P28-Hind-III, and cloned at the Sal-I and Hind-III sites of a pBSK vector. Thereafter, a luciferase gene (Luciferase-pcDNA3, Plasmid #18,964, Addgene) was cloned at Hind-III and BamHI sites of a vector containing respective promoter sequence. The luciferase gene was amplified from luciferase gene containing plasmid using the primers P25-Hind-III and P26-BamHI. A 3′UTR (~ 627 bp) from the 3′end of the *BMR1_03g03490* gene was cloned at BamHI and NotI restriction sites. This construct was named as plasmid **T5.**

All the Plasmid constructs were isolated using Qiagen Midi-prep kit, concentration and purity was determined using OD value at 260 and 280 nm. The relative promoter activity was quantified by measuring the luciferase units in transfected parasites.

### Transient transfection

Briefly, 100 μl of *B. microti* (grey strain, ATCC 30221D) infected blood (~ 10% parasitemia) was collected from infected mice in a 1.5 ml tube with 10 µl of heparin (10,000 units/ml). The infected blood was centrifuged at 1,000 rpm at room temperature and washed once with the RPMI 1,640 media (Gibco, Thermo Fisher Scientific, India). Approximately, ~ 1 × 10^6^ infected RBCs were mixed with the 90 µl of the transfection buffer (VAPA-1002, Lonza) and 10 µg of construct DNA. The electroporation was performed in a 1 cm cuvette using U033 protocol, with the help of Nucleofector-2B device (Lonza, Switzerland). A 100 µl complete RPMI media was immediately added to the transfected parasites and transfected parasites were injected into the C57BL/6 J mice via intravenous injection. In a control experiment, parasites were transfected with a 10 µg of control plasmid DNA without GFP or any other marker.

#### Fluorescence microscopy

Two days post-transfection a drop of blood was collected in a 1.5 ml tube from the tail of mice previously injected with transfected parasites. The blood was diluted in phosphate buffer saline (PBS) in a ratio of 1:50. The 10 µl of the diluted blood was mounted on a glass slide with a glass cover slip. The transfected parasites were analyzed to detect GFP expression using Axio-Imager-M2 fluorescence microscope (Zeiss, Germany).

### Construction of a plasmid DNA for stable transfection in *B. microti* parasite

We used a double homologous recombination strategy to insert a GFP expression cassette into the genome of *B. microti* parasite. A GFP expression cassette was inserted to replace a 200 bp region (468,280–468,503) present in an intergenic sequence between *Bmr1-11,102,355* and *BMR1_01G01285* parasite genes. The construct for stable transfection contains a ~ 820-bp fragment from the upstream region of 200 bp of intergenic sequence (468,280–468,503) and was amplified by PCR using primer P17 and P18. This fragment was cloned between the KpnI and XhoI sites of the GFP/mCherry construct (plasmid T3), which was used in the transient transfection. A ~ 716-bp fragment from the downstream region of a 200 bp in intergenic sequence (468,280–468,503) was amplified by the PCR using primers P19 and P20. The fragment was cloned between NotI and SacI restriction sites in a GFP/mCherry construct plasmid as described previously in 1.3 method section. The region was selected because the size of an intergenic sequence between these two genes is ~ 4.6 kb and the deletion of 200 bp nucleotides in the middle of this intergenic sequence is very unlikely to interfere with the expression of adjacent genes in the parasite. Analysis of the 200 bp DNA including bioinformatics analysis shows it does not contain a conserved motif or a promoter sequence. The GFP expression cassette for stable transfection is same as described in our transient transfection experiments. This construct was named as plasmid **T6.** 10 µg of plasmid T6 was linearized by restriction digestion using SapI enzyme. The linearized construct DNA was precipitated and suspended in 10 µl of sterile water.

### Stable transfection

The 100 µl of blood was collected from *B. microti*-infected mice (10–20% parasitemia) into a 1.5 ml Eppendorf tube containing heparin, washed twice with incomplete RPMI media. Human T-cells nucleofector kit (VAPA-1002, Lonza, Basel, Switzerland) was used for the parasite transfection. First, 18 µl of solution ‘A’ of the kit and 82 µl of solution ‘B’ of the kit were mixed in a tube and kept on the ice. The 10 µg of the linearized construct was added to the solution. Next, 20 µl of packed RBC with 10% parasitemia were added to the transfection mixture per transfection. The transfection was performed using the manufacturer’s U033 program in a Nucleofector-2B machine (Lonza, Basel, Switzerland) and 100 µl of incomplete RPMI (Gibco, Thermo Fisher Scientific, India) was added to the transfected parasites. The transfected parasites were immediately injected (Intravenous) into the mice. In a control experiment a plasmid without homologous arms and without a reporter gene was used as a control. After 48 h, the transfected parasites were analyzed by fluorescence microscopy to visualize GFP expressing parasites. The GFP expressing parasites were sorted by FACS and immediately injected into a mouse to enrich the transfected parasites.

#### Fluorescence activated cells sorting (FACS) and single parasite dilution cloning

The single parasite population was achieved through the limiting dilution cloning of transfected parasites. A small amount of blood (~ 50 µl) was collected from the tail of infected mice and diluted in RPMI media (Gibco, Thermo Fisher Scientific, India) to 1 × 10^6^ cells per ml. GFP or mCherry, expressing parasites were sorted by BD FACS Aria. The sorted parasites were diluted in RPMI to obtain 5 parasites per ml and 200 µl (one parasite) of the diluted media was injected into each mouse in a total of 10 mice. The parasitemia of injected mice was observed every day and mice were sacrificed when parasitemia reached to ~ 10%.

#### Diagnostic PCR and southern blot analysis of transfected parasites

Genomic DNA of the transfected clonal parasites was isolated by blood Mini kit for gDNA isolation (Qiagen, Germany). Integration specific diagnostic PCR was performed for 5′ integration events using primers P21 and P22. Similarly, 3′ integration specific diagnostic PCR was performed using p23 and P24 primers. These pairs of primers will give PCR product only on the integrant gDNA and not in wild type (WT) gDNA. The size of the expected PCR product was 1.3 kb for the 5′ integration site and 1.5 kb for the 3′ integration site. To further confirm integration, a Southern blot was performed to determine the integration of the reporter genes at the right locus in the parasite. Briefly, the gDNA of the transgenic and WT parasite was digested with Eag-I restriction enzyme overnight and the resulting DNA fragments were separated using a 0.8% agarose gel run at a 50 V. The DNA was then transferred to a positively charged nylon membrane and cross-linked with UV. The membrane was incubated for about 3–4 h in a pre-hybridization buffer at 50 degree Celsius in a hybridization chamber. The membrane with DNA was further processed for hybridization with a DIG labeled GFP probe at 50 degree Celsius for 12 h. The membrane was washed three times with an appropriate washing buffer that removes non-specifically bound probe but leaves the DNA-bound probe. An anti-DIG antibody was used at 1:5,000 dilutions to detect the probe. The blot was developed using the CSPD substrate, which is a chemi-luminescence substrate. A band of 10.5-kb is expected in transgenic parasite by the GFP probe. The probe was amplified by PCR using primers P3 and P4 and labeled using a DIG-labeling kit (Roche, Basel, Switzerland,), according to the manufacturer’s protocol.

### Growth analysis of wild type and stably transfected parasite

A growth curve analysis of the transgenic parasites (*B. microti-*GFP-mCherry) was performed by blood-stage parasitemia examination over 8–9 days. The mice were injected with 10,000 transgenic or WT parasites in a group of 10 mice each. Parasitemia were determined for both the wildtype (WT) and the transfected parasites. The blood-stage parasitemia was determined by the Giemsa method. In this method, first a thin smear of blood is made on the glass slide, fixed with methanol and then stained with Giemsa stain for about 15 min. The parasite numbers, in the blood of infected mice, were determined by bright field 100 × objective microscope. Parasitemia was measured daily. The pre-patent period, i.e. the first appearance of the parasite in the blood of infected mice, was determined in case of WT and transgenic parasites.

## Supplementary information

Supplementary file1
