# Supplementary material for: Establishment of a stable transfection method in Babesia microti and identification of a novel bidirectional promoter of Babesia microti
Source: Sci Rep. 2020 Sep 24;10:15614. doi: 10.1038/s41598-020-72489-3 (PMC7515924; doi:10.1038/s41598-020-72489-3)
Supplement: Supplementary file 1 — Supplementary file1 [file 41598_2020_72489_MOESM1_ESM.doc]

**Establishment of a stable transfection method in *Babesia microti* and Identification of a novel bidirectional promoter of *Babesia microti***
Dabbu Kumar Jaijyan^1^**^*^**, Kavitha Govindasamy^2^ **^* #^**, Jyoti Singh^1^, Shreya Bhattacharya^1^, Agam Prasad Singh^1^ **^#^**

1 National Institute of Immunology, Aruna Asaf Ali Marg, New Delhi, India

2 MJKS Research LLC, Edison, New Jersey, USA

**Original Blots of figure 4 B and 4C.**

**
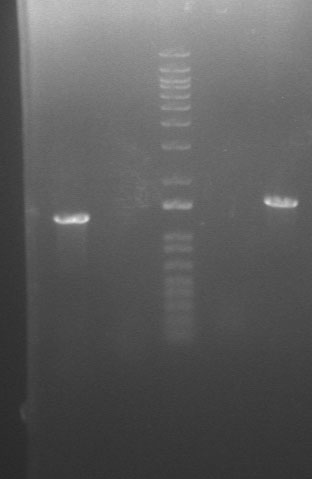
**

**Figure legend 4B:** Diagnostic PCR. The primer P21 and P22 is specific for the 5’ integration event and it should not give any PCR product on WT gDNA. The primer P23 and P24 is specific for the 3’ integration event. A PCR product of right size in the diagnostic PCR confirmed the integration of GFP-mCherry cassette at the right locus in *B. microti* parasite.


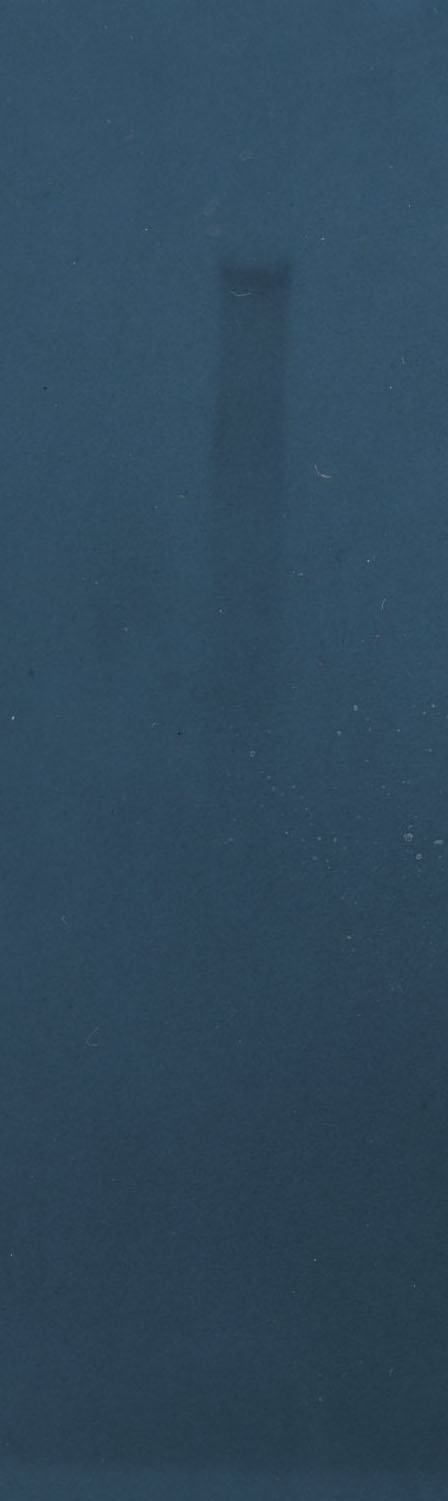


**Figure legend 4C:** Southern blot analysis of transfected parasites. The DIG labeled GFP probe was used to detect the integration event in the digested gDNA. A band of ~10.5 kb was detected in the transgenic *B. microti* parasite. Wild-type parasites do not have the gene for GFP hence no bands are expected.
